# Antibiotic susceptibilities of indicator bacteria *Escherichia coli* and *Enterococci* spp. isolated from ducks in Morogoro Municipality, Tanzania

**DOI:** 10.1186/s13104-018-3201-4

**Published:** 2018-01-31

**Authors:** Henry D. Kissinga, Festo Mwombeki, Khadija Said, Abdul A. S. Katakweba, Hezron E. Nonga, Amandus P. Muhairwa

**Affiliations:** 1 Pest Management Centre, Sokoine University of Agriculture, P.O. Box 3010, Morogoro, Tanzania; 2 Department of Veterinary Medicine and Public Health, Sokoine University of Agriculture, P.O. Box 3021, Morogoro, Tanzania

**Keywords:** Ducks, Duck droppings, Use of antibiotics, *E. coli*, *Enterococci*, Antibiotic resistance

## Abstract

**Objective:**

To estimate the prevalence of antibiotic resistance in indicator bacteria *Escherichia coli* and *Enterococci* isolated from duck faeces in Morogoro Municipality, Tanzania.

**Results:**

*Escherichia coli* and *Enterococcus* isolation rates from ducks faeces were 91 and 100% respectively. The prevalence of antibiotic resistance of *E. coli* and *Enterococcus* was 70.3 and 42%, respectively. *E. coli* resistant to four antibiotics were 28 (30.8%) and showed high resistance to ampicillin (81.3), tetracycline (75.8) and trimethoprim–sulphamethoxine (62.3). Multiple antibiotic resistance of *Enterococcus* were more than 65%. High resistance rates shown by *Enterococcus* were observed in rifampin (62%), ampicillin (62%) and tetracycline (42%). Almost all farmers (92.3%) left their ducks to scavenge for food around their houses. Antibiotics used in animal treatments were oxytetracyclines, sulfonamides, penicillin dihydrostreptomycin while in humans were tetracycline, ampicillin, and amoxicillin.

**Electronic supplementary material:**

The online version of this article (10.1186/s13104-018-3201-4) contains supplementary material, which is available to authorized users.

## Introduction

Antibiotic resistance is an emerging world health problem since many bacteria have developed resistance to commonly used antibiotics and the probable reasons are indiscriminate uses of these medicines. In Tanzania, antibiotics are used for the treatment of diseases in humans and animals and as prophylaxis and growth promoters in livestock [1]. Uses of antibiotics in Tanzania are not controlled; patients and farmers can access any kind of these medicines over the counter without prescriptions [1]. In human medicine, the emergence of resistant bacteria is correlated to over-prescription of antibiotics [2]. The ever-increasing uses of antibiotics are a problem in human medicine and livestock, selection pressure has further increased the advantage of the bacteria to acquire, maintain and disperse resistant genes to other bacterial populations [3].

Antibiotic resistance has been studied using indicator bacteria like *Escherichia coli* and *Enterococcus* spp. which showed good results [4, 5]. A study on antibiotic resistance in ducks in Tanzania showed high rate of multidrug-resistant *Campylobacter jejuni* [6], however, no attempts were done to explain the possible predisposing factors neither the resistant in indicator bacteria. Other studies elsewhere on *E. coli* from ducks showed also high rates of multidrug resistance [7, 8]. *Enterococci* spp. has been reported to show high resistance to vancomycin [9]. Despite the increase of interest in *E. coli* and *Enterococci* spp. antibiotic resistance studies worldwide, there is no such studies in Tanzania. This study determined the prevalence of antibiotic resistance of *E. coli* and *Enterococci* spp. isolated from the scavenging ducks (free ranging) that are clinically healthy in Morogoro Municipality, Tanzania.

## Main text

### Methods

#### Study area and duck sampling

This cross sectional study was conducted in Morogoro Municipality between November 2014 and March 2015 where 100 Muscovy ducks (*Carina moschata*) were sampled from 26 backyard flocks [10]. Study ducks were randomly selected from each flock where 5–10% of the flock size was sampled [11]. The sampled birds included 86 females and 14 male ducks. Before sampling, basic information on management, feeding, uses and disposal of unused antibiotics were gathered using a questionnaire (Additional file 1). The selected ducks were manually restrained and a sterile cotton swab was inserted into cloaca to collect faecal materials. The samples were stored in cool box with ice pack and transported to the laboratory at Sokoine University of Agriculture for analysis. For consistency, two researchers after were trained by the laboratory technologists on swab sampling from birds did the sampling to all the 100 ducks.

#### Isolation and identification of *E. coli* and *Enterococci* spp.

Faecal samples were plated on MacConkey agar (Oxoid, Basingstoke, UK) and incubated for 24 h under aerobic condition for isolation of *E. coli* [12]. Suspect colonies were further identified based on colonial morphology, Gram stain and biochemical tests [12]. For isolation of *Enterococci* spp., samples were plated on Slanetz-Bartley agar (Oxoid, Basingstoke, UK) and incubated for 48 h under aerobic condition and identification based on colonial morphology and biochemical tests [9]. During isolation and identification, *E. coli* ATCC25922 and *Enterococcus faecium* ATCC 2912 were included as control strains.

#### Antibiotic susceptibility

All the *E. coli* and *Enterococci* isolates were tested for antibiotic susceptibility using disk diffusion method and interpreted according to the CLSI [13]. Antimicrobial susceptibility testing was performed to some antibiotics which are commonly used in human medicine in Tanzania. Four antibiotics for *E. coli* were tested at indicated concentrations: ampicillin 10 µg, cefotaxime 30 µg, trimethoprim–sulphamethoxine 25 µg and tetracycline 30 µg. *Enterococci* were tested against ampicillin 10 µg, erythromycin 15 µg, rifampin 5 µg, tetracycline 30 µg and trimethoprim–sulfamethoxine 25 µg. All the antibiotic discs were from Oxoid, Basingstoke, UK.

#### Ethical considerations

The permission to carry out this study was granted by the Morogoro Municipal Livestock Officer and ethical approval for the study was given by the Ethical Committees of Sokoine University of Agriculture, Tanzania. Verbal consent was obtained from each of the farmers prior to sampling.

### Results

Out of 100 faecal samples cultured 91% had *E. coli* of which 64 (70.3%) showed resistance to different antibiotics tested (Table 1). Multiple antibiotic resistances shown by *E. coli* were: 28 (30.8%) isolates to four antibiotics, 35 (38.5%) to three antibiotics, 13 (14.3%) to two antibiotics, 11 (12.1%) isolates to one antibiotic. *Enterococcus* was isolated from all the samples and 42% showed resistance to different antibiotics tested. Five percent of *Enterococcus* isolates were resistant to all antibiotics, 13% to four antibiotics, 21% to three antibiotics, 26% to two antibiotics and 28% to one antibiotic tested. Results on duck management and their possible access to antibiotic residues are summarized in Table 2. Of the 26 farmers interviewed, 92.3% managed ducks extensively where they accessed to dumping places. No administration of veterinary drugs was reported to ducks but respondents reported to treat other animals by using tetracyclines/oxytetracycline.

**Table 1:** Prevalence (%) of antibiotic resistance of *E. coli* (n = 91) and *Enterococcus* spp. (n = 100) isolated from duck

Antibiotic tested	Number of resistant isolates (%)	
	*E. coli*	*Enterococcus* spp.
Ampicillin	74 (81.3)	62 (62)
Cefotaxime	57 (62.3)	–
Trimethoprim–sulphamethoxine	69 (75.8)	18 (18)
Tetracycline	54 (59.3)	42 (42)
Erythromycin	–	19 (19)
Rifampin	–	62 (62)

**Table 2:** Duck management and their possible access to antibiotic residues (n = 26)

Parameter assessed	Response category	Number (%)
Duck flock size	5–50	21 (80.8)
	51–120	5 (19.2)
Duck management (feeding system)	Extensive	15 (57.7)
	Intensive given maize bran and kitchen wastes	2 (7.7)
	Both	9 (34.6)
Ducks scavenging areas	Around homestead areas and neighbouring households areas	21 (80.8)
	Around homestead areas	5 (19.2)
Number of dumps the ducks access during scavenging	One dumping place	6 (23.1)
	Two dumping places	9 (34.6)
	Three dumping places	9 (34.6)
	Four dumping places	2 (7.7)
Duck treatment	Yes	0 (0.0)
	No	26 (100.0)
Keeping other animals apart from ducks	Yes	24 (92.3)
	No	2 (7.7)
Treatment of other animals	Yes	13 (50.0)
	No	13 (50.0)
Antibiotic used in treatment of other animals	Tetracycline/oxytetracyclines	4 (15.4)
	Sulfonamides	3 (11.5)
	Oxytetracyclines, sulfonamides, penicillin dihydrostreptomycin	2 (7.7)
	Other antibiotic not known	4 (15.4)
	No treatment	13 (50.0)
Commonly used antibiotics in humans	Tetracycline	4 (15.4)
	Ampicillin	2 (7.7)
	More than one antibiotic (tetracyclines, ampicillin, amoxicillin, sulphonamides)	8 (30.8)
	Other antibiotic not known	12 (46.2)
Drug containers and unused medicine disposal system	Dump	13 (50.0)
	Latrines	7 (26.9)
	Burn	6 (23.1)

### Discussion

The current study found that the *E. coli* isolation rates was 91% while all ducks haboured *Enterococci* and the isolates had high rates of multi-antibiotic resistance. This study shed light that the indicator bacteria from free scavenging ducks in Tanzania can be used in studies of antibiotic resistance. This suggests that the problem of antibiotic resistance is widespread even to bacteria isolates from animals which do not receive antibiotic therapy. This has a public health impact since ducks scavenge for the feed around homestead areas and shed their faecal droppings all over and the resistant bacteria can find their way into the food chain and affect humans or the resistant gene can be transferred to pathogenic bacteria [14].

The estimated prevalence of antibiotic resistance in *E. coli* isolated during this study is comparable to resistance reported in Malaysia [15]. In Nigeria, a low prevalence of 8.6% has been reported probably due to using different antibiotics like ofloxacin, nalidixic acid and gentamycin [7]. The high prevalence of antibiotic resistance observed during this study probably ducks are constantly exposed to antibiotic residues from animal and human discharges because of wide uses [1, 16]. Studies show that antibiotics used in the food animals are poorly absorbed in the gut and the parent compound are excreted in faeces [17–19]. This is supported by several studies that have reported high levels of antibiotic residues in sewage and animal wastes [20–24]. Therefore, the normal flora in scavenging ducks are constantly exposed to antibiotic residues which makes them develop resistance as it was observed during this study.

The *E. coli* resistance to ampicillin was the highest (81%) compared to other antibiotics used in the study similar to previous report in Slovakia where the resistance recorded was 87.8% [25]. Generally, studies show that 60–70% of *E. coli* isolates have developed resistance against ampicillin [18, 26]. This may be caused by too much uses of ampicillin in human practice which its residues are potential sources of environmental contaminations where ducks scavenge for feed. In case of sulphamethoxine, the resistance recorded was 76% as previously recorded by Van Tuat [27]. The antibacterial resistance shown by tetracycline in this study was 59% while in Slovakia was 37.5% [8] and that of Nigeria was 75% [7]. Interestingly, *E. coli* also showed antibacterial resistance to cefotaxime at 63%, an antibiotic not commonly used in the treatment of the animals in Tanzania. Ducks may access cefotaxime residues in dumps where human faeces and disposed drug remains are found (Table 2). However, high resistance to cefotaxime has been observed with bacteria isolated from different health facilities sewerage [28].

Multiple resistances of *E. coli* to more than one antibiotic were also observed in the study. The findings were too high when compared with the study conducted in Vietnam where multiple antibiotic resistance to *E. coli* was 16.7% to four antibiotics, to three antibiotics 20% and to two antibiotics was 23.3% [27]. This occurrence of multiple drugs resistance suggested that livestock played a role as reservoirs of resistant bacteria for environmental contamination [29]. It has been found that bacterial populations isolated from the gut of animals exposed to antibiotics were found to be five times more likely to be resistant to any given antibiotic. The non-resistant bacteria normally acquire the extra-chromosomal antibiotic resistance plasmids (R-plasmids) from the resistant ones when are in close contact [18, 26, 30]. The observed resistance may be contributed by irrational use of antibiotics which discharges a substantial amount of residues to the environment. Other factors which cause the development of resistance could be the easy availability and rampant use of broad-spectrum antibiotics in the presumptive treatment of infections even in health facilities. Lack of enforcement of regulations on antibiotic use as a part of infection control programmes could have influenced the pattern of resistance results to a considerable degree.

*Enterococci* spp. are natural commensals of the human and animal gut. All the study ducks had *Enterococci* spp. in their gut contents which showed 42% resistance rate to different antibiotics tested. Nevertheless, findings from this study showed high resistance in rifampin (62%), ampicillin (62%) and tetracycline (42%). Multidrug resistance was also observed in more than 65% of the isolates. As earlier stated, the observed resistance of the bacteria to different antibiotics tested in absence of uses of the medicines in ducks could be due to residues discharged to the environment where ducks scavenge can easily get exposed to antibiotics and other resistant bacteria [20–24]. When resistant bacteria are in contact with the nonresistant ones, exchange of genetic materials can take place and the resistant gene can be acquired. Elsewhere, studies have reported that *Enterococci* spp. isolated from ducks showed resistance against macrolides and lincosamide antibiotics [31–33]. Nevertheless, *Enterococci* spp. is intrinsically resistant to several antibiotic groups including cephalosporins and aminoglycosides. In addition, many strains harbour transmissible genetic elements for acquired resistance to various antibiotics like tetracycline [34]. Other antibiotic resistance studies in *Enterococci* spp. isolated from ducks have been carried out using vancomycin antibiotic [9]. Further susceptibility studies of *Enterococci* spp. to vancomycin is suggested in future since the antibiotic is nowadays a common drug in human medicine in Tanzania.

The questionnaire study further supports the laboratory observation on high resistance rate shown by *E. coli* and *Enterococcus* spp. It was observed that antibiotics are widely used in the treatment of animals and humans. Regardless of non-uses of veterinary drugs in dusks, the extensive scavenging management system exposes them to the environment with antibiotic residues and other bacteria from animals and humans, which have been on antibiotics. This is supported by the findings that the observed resistances were to antibiotics that are commonly used in humans and animals (Table 2). Resistance bacteria have been isolated from the variety of sources including sewage discharge and the environment [35–37]. Drug remains and containers were haphazardly disposed of in the household surrounding environments which may expose ducks to the antibiotic. Therefore, the intensive management system of ducks can help to minimize the unnecessary exposure of birds to antibiotic residues.

### Conclusion

Generally, antibiotic resistance shown by *E. coli* and *Enterococcus* spp. in this study was high. This shows the role played by livestock and chicken which are constantly on antibiotics as potential primary sources of antibiotic resistance genes to bacteria isolated from ducks. Isolating multi-antibiotic resistant *E. coli* and *Enterococcus* spp. from ducks serve as potential carriers of antibiotic-resistant strains to humans and poses a serious problem to the public health.

### Limitations

This study used only 100 duck samples collected from Morogoro Municipality which limits generalization of the results to be used as representative on the status of antimicrobial resistance with *E. coli* and *Enterococcus* spp. in ducks in Tanzania. Also the numbers of antibiotic tested were few. Testing for antibiotic susceptibility of bacteria using disk diffusion test method may be inferior to broth microdilution methods. Also, the study did not determine the resistance genotypes in *E. coli* and *Enterococcus* spp. which were resistant to different types of antibiotics. Nevertheless, presence of other animals on-site where duck sampling was done acts as confounding factors on the resistance rates observed in bacteria isolates from ducks.

## Additional file

**Additional file 1.** Questionnaire on duck management, antibiotic uses and disposal in Morogoro Municipality, Tanzania. The questionnaire was used as a data collection tool from small scale duck farmers in Morogoro Municipality, Tanzania.

## Abbreviations

UK: United Kingdom
CLSI: Clinical and Laboratory Standards Institute
µg: microgram
spp: species
SUA: Sokoine University of Agriculture
COSTECH: Commission for Science and Technology
