# Supplementary material for: Antibiotic susceptibilities of indicator bacteria Escherichia coli and Enterococci spp. isolated from ducks in Morogoro Municipality, Tanzania
Source: BMC Res Notes. 2018 Jan 31;11:87. doi: 10.1186/s13104-018-3201-4 (PMC5793419; doi:10.1186/s13104-018-3201-4)
Supplement: Supplementary file 1 — Additional file 1. Questionnaire on duck management, antibiotic uses and disposal in Morogoro Municipality, Tanzania. The questionnaire was used as a data collection tool from small scale duck farmers in Morogoro Municipality, Tanzania. [file 13104_2018_3201_MOESM1_ESM.doc]

**Questionnaire: Antibiotic susceptibilities of indicator bacteria *Escherichia coli* and *Enterococci* spp. isolated from ducks in Morogoro Municipality, Tanzania**

**A: DUCK OWNER DEMOGRAPHIC DATA**

1. Date of interview (dd/mm/yy)………………………………………………………………….
2. Interviewee’s names (Optional)………………………………………………………………..
3. Sex…………………………………………….……. Age (years)…………………………..
4. Ward……………………. Street……………………………………………………………….

**B: GENERAL DUCK MANAGEMENT AND USES OD ANTIBIOTICS**

1. How many ducks do you own?....................................................................................................
2. What management system do you manage your ducks ?
3. Intensive management system
4. Extensive management system (scavenging for feed)
5. Semi-intensive (mixed system intensive and extensive) management system
6. Do you feed your ducks? Yes……………..No………………
7. If yes, which feed to you give to your ducks…………………………………………..
8. If the ducks are extensively managed, where do they scavenge for feed?
9. Are there dumping areas in your homestead? Yes……………..No………………
10. If answered yes, how many dumping areas are around your homestead? ………………
11. Do the ducks sometimes use the dumps as sources of feed? Yes……………..No……………
12. Do you keep other animals apart from ducks? Yes……………..No………………
13. If answered yes, which other animal do you keep?.......................................
14. Have your ducks ever been sick? Yes……………..No………………
15. If answered yes, what diseases have ever affected your ducks?..........................................................................................................................................
16. Do you treat ducks when are sick? Yes……………..No………………
17. If answered yes, which medicine do you use to treat ducks? Please list them…………………
18. Do the other animals you keep get sick? Yes……………..No………………
19. If answered yes, do you treat the other animals with medicines? Yes………..No……………
20. If answered yes, which medicine do you use to treat the other animals?

Please list them……………………………………………………………………………………

1. Are antibiotics used in treatment of sick family members in your household? Yes……No…..
2. If answered yes, which medicines are used in treatment of sick individuals in your family?

Please list them…………………………………………………………………………………….

1. Where do you dispose unused antibiotics? …………………………………………………….
2. Where do you dispose empty medicine containers?...................................................................
